# A Study on the Rheological Properties of Recycled Rubber-Modified Asphalt Mixtures

**DOI:** 10.1155/2015/258586

**Published:** 2015-01-27

**Authors:** Murat Karacasu, Volkan Okur, Arzu Er

**Affiliations:** ^1^Department of Civil Engineering, Eskisehir Osmangazi University, 26480 Meselik, Eskisehir, Turkey; ^2^Department of Civil Engineering, Akdeniz University, 07058 Antalya, Turkey

## Abstract

Using waste rubber in asphalt mixes has become a common practice in road construction. This paper presents the results of a study on the rheological characteristics of rubber-modified asphalt (RMA) concrete under static and dynamic loading conditions. A number of static and dynamic creep tests were conducted on RMA mix specimens with different rubber sizes and contents, and a series of resonant column tests were conducted to evaluate the shear modulus and damping values. To simulate the stress-strain response of traffic-induced loading, the measurements were taken for different confining pressures and strain levels. The results of the study indicated that rubber modification increases stiffness and damping ratio, making it a very attractive material for use in road construction. However the grain size of the rubber is very important. Although RMA may cost up to 100% more than regular asphalt, the advantages it brings, such as an increased service life of the road and proper waste utilization contributing to a more sustainable infrastructure, may justify the added cost.

## 1. Introduction

Asphalt concrete is the leading paving material for roads and runways. Understanding the characteristics of the asphalt being used in a project is important to ensure long-term performance and stability. Various environmental conditions and traffic-induced loadings must be taken into account in the design stage. Sometimes the design does not result in acceptable, safe, and sound usability due to early deterioration. Several factors contribute to deterioration, such as the quality of materials and construction, traffic loading on the road, road geometry, and environmental conditions. In general, most of the deterioration is due to rutting or bottom up fatigue and thermal cracking. Enhancing the pavement life is possible with some modifications if possible factors causing deteriorations are taken into consideration during the design stage.

Plastomeric polymeric materials, such as polyethylene (PE) and polypropylene (PP), have evoked considerable interest among engineers and manufacturers for use in road paving modification because of their viscoelastic properties and good adhesion to mineral aggregates [1–4]. The main purpose of using polymers in asphalt concrete is to increase binder stiffness at high service temperatures and reduce stiffness at low service temperatures [5–7]. Polymers which are used for the modification of asphalt concrete can be divided into three main categories: thermoplastic elastomers, plastomers, and reactive polymers. Thermoplastic elastomers are apparently capable of high elastic response characteristics and therefore resist permanent deformation by stretching and recovering their initial shape on the modified binder layer, whereas plastomers and reactive polymers modify asphalt by forming a tough, rigid, three-dimensional network to increase stiffness and decrease deformations [8, 9]. Because of its suitability in these conditions, one of the leading polymer modifiers for bitumen among the larger group of copolymers is styrene-butadiene-styrene (SBS) block. SBS is a synthetic hard rubber copolymer that is used in applications where durability is important and is often substituted in part for natural rubber based on the comparative raw materials' costs [10–15]. It belongs to the group of copolymers called block copolymers, in which the backbone chain is made up of three segments. The first is a long chain of polystyrene, the middle is a long chain of polybutadiene, and the last segment is another long section of polystyrene.

SBS is a cost-effective material that is used to stretch dwindling natural rubber resources, especially in tire manufacturing. However the disposal of used automobile tires has caused many environmental and economic problems. The annual global production of used tires estimated 17 million tonnes in which China, European Union Countries, USA, Japan, and India lead the way to produce the largest amounts of tyre [16]. A small percentage of these tires are recapped or reused as lower-quality rubber, but around 80% of these tires are accumulated in dumps, posing health hazards and adversely impacting the environment [17].

Due to its elastic nature, crumb rubber can be used in road construction to improve deformation resistance. Rubber-modified asphalt (RMA) is a bituminous mix consisting of blended aggregates, recycled crumb rubber, and bitumen. It is found out that rubber structure is the most important factor affecting elastic properties. Crumb rubber has a granular texture and ranges in size from a very fine powder (<0.1 mm) to coarse particles (>5 mm). Choubane et al. [18] published the findings from a ten-year study of RMA surfaces and concluded that crumb rubber increases overall strength and reduces surface rutting. RMA has been known to improve the rheological properties at low and high temperatures and provides a lifespan that is up to three times longer than conventional asphalt [19]. There are also other reasons why rubber is useful in both highways and railways, such as decreasing thermal instability, increasing resistance to low-temperature cracking, reduction of noise levels, and reduction of vibrations generated by heavy axle loads [20, 21].

The focus of the test program was to examine the performance of asphalt concrete when mixed with crumb rubber as aggregate. Same type rubber in different sizes and shapes was added to the mix without any modification to the bitumen content. The study concentrated on the behavior of admixture under cyclic loadings. The digestion mechanism of the rubber sizes is not evaluated in this paper but should be considered in the future research.

## 2. Experimental Procedure

**Figure 1:**
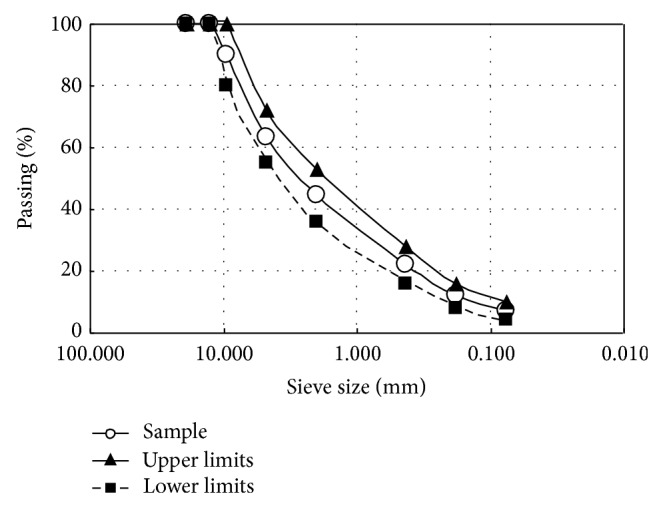
Aggregate grading curves for asphalt mixtures compared with the current GDTH.

The appropriate aggregate gradation for hot-mix bitumen was designed according to the technical specifications of the General Directorate of Turkish Highways (GDTH) 2006 [22]. The aggregates have a mean grain size (*D*_50_) between 0.30 and 3.0 mm and a coefficient of uniformity (*C*_*u*_) between 2.0 and 3.0. The margins of the GDTH and the prepared grading curves are given in Figure 1. The physical characteristics of the aggregates and properties of the mix design are given in Tables 1 and 2, respectively.

**Figure 2:**
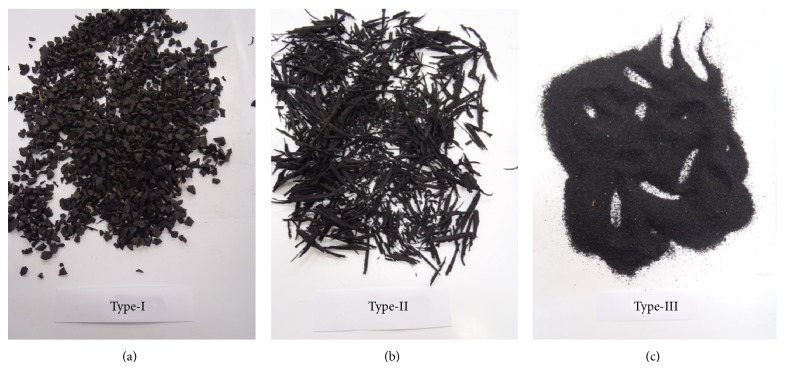
Tire rubber after the shredding and grinding: (a) Type-I, (b) Type-II, and (c) Type-III process.

**Figure 3:**
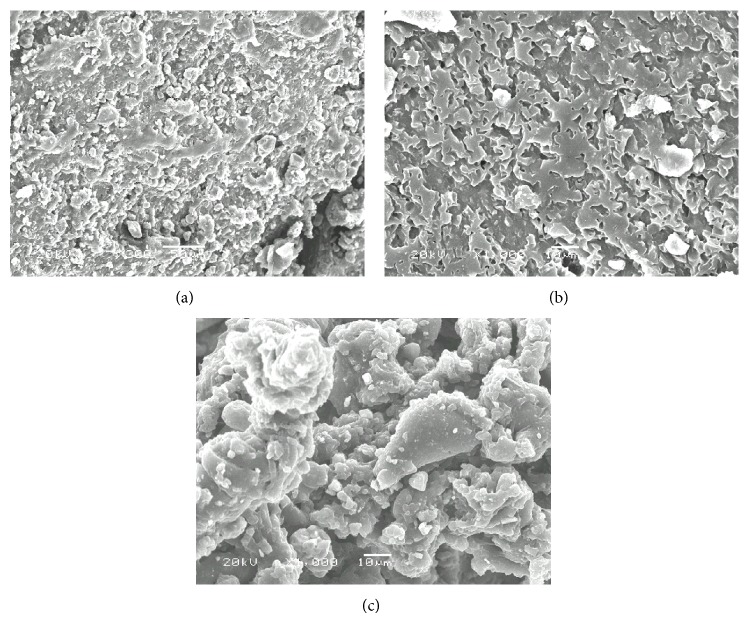
Scanning electron micrographs of waste tire: (a) Type-I, (b) Type-II, and (c) Type-III.

To determine the optimal bitumen content, Marshall stability tests and flow tests are performed according to the ASTM D 1559-76 specifications. The optimum bitumen ratio was found to be 4.89% for the 50/70 penetration grade. The crumb rubber selected for this investigation was produced from recapping automobile tires. The bitumen was modified with three different sizes of tire rubber by-products: Type-I (granulated tire rubber that passes a #3/8 sieve), Type-II (5–15 mm pieces of chipped tire rubber), and Type-III (powdered tire rubber that passes a #40 sieve). All rubbers were obtained by shredding and grinding the tire after removing the fabric and steel belts. Photographs and SEM images compares the crumb rubber gradation and surface texture for three types in Figures 2 and 3, respectively.  Table 3 shows the physical properties of the virgin binder. The characteristics of the RMA depend on the concentration amount and the polymer type in which the polymer is mixed in concentrations of approximately 0.2–1.0% by weight of aggregates. Higher concentration mixes of polymers can be less economical and may also cause problems related to the material properties [1].

Rubber contents of 0.2%, 0.4%, 0.6%, 0.8%, and 1.0% by weight of aggregates were blended with bitumen for each rubber size at a mixing temperature of approximately 160°C. To verify the repeatability of the tests, three specimens are prepared using an identical procedure (premixing the rubber with bitumen using a mixer at 500 rpm for 2 minutes) from each mix. Visual observation of the flowable bitumen produced revealed that the crumb rubber was well mixed in the fluid state without clumping. A total of 90 modified specimens were used to determine the optimum rubber amount for each type. In addition, 3 Control specimens with no additives were prepared for comparison with the modified specimens.

**Figure 4:**
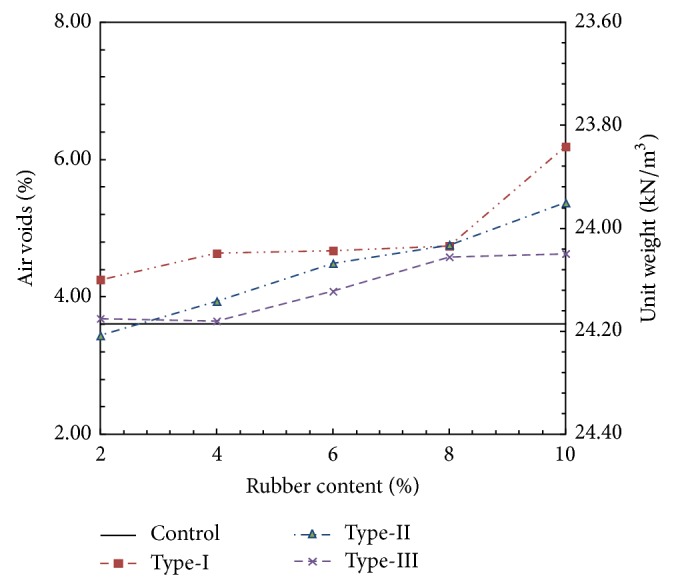
Variation in air voids and unit weight with respect to rubber content.

**Figure 5:**
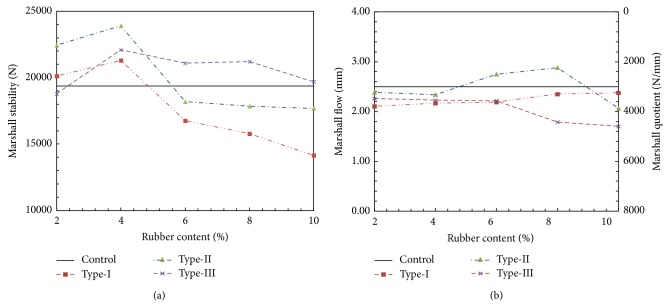
Effect of rubber content on (a) Marshall stability and (b) Marshall flow and quotient.

The average values obtained from the Marshall tests are summarized in Figures 4 and 5. The solid line in each figure shows the boundary value for the Control specimen.  Figure 4 shows the variation in air voids and unit weight against the rubber content. Note that increasing the rubber content tends to increase the void ratio. Furthermore, the void ratio at a given rubber content tends to increase with the increasing size of the rubber particles. This tendency is also coincident with the unit weight.

Figure 5(a) shows the effect of rubber content on Marshall stability, which has a general tendency to decrease as rubber content increases. The initial increase of the Marshall stability with increase of rubber content for Type-III can be attributed to the size effect of the rubber. The Marshall quotient has a tendency to decrease with increasing rubber content for all modified specimens except Type-III, where the Marshall quotient value is almost the same with increasing rubber content (Figure 5(b)). It is seen that the addition of 0.4% rubber has the most significant impact on the Marshall characteristics of the specimens, and these contents are therefore used for the subsequent creep and resonant column tests.

## 3. Tests

For practical purposes, the method of load application for creep testing in a laboratory environment can be classified into two types: static and dynamic loading. The static loading condition simulates a heavy vehicle, such as a truck, standing on a pavement specimen, and applying a static stress by waiting at a red light. Static creep tests were performed in the laboratory to evaluate the response of an asphalt specimen for such a condition. In a static creep test, the measured data are the deformation time, which is the length of time the pavement can resist the static load until flow occurs. On the other hand, repetitive loading simulates the driving of a heavy vehicle over a specimen of pavement. This condition can be reproduced by applying a dynamic load to an asphalt specimen. The output data for a repetitive test are the number of load cycles that the pavement can tolerate before it fails. This test is destructive, so the asphalt specimen can be tested only once.

For both tests, the specimens were prepared according to EN12697-25A specifications, with a diameter of 100 mm and height of approximately 63.5 mm. The testing temperature was set to 50°C, and the specimens were kept in a climatic cabinet for 24 hours.

### 3.1. Static Creep Test

To enable perfect coupling between the specimen and top platen a static axial stress of *σ*_*s*_ = 5 kPa was applied for ten minutes. After the application of prestress, the specimens were axially loaded to a value of 500 kPa in approximately 1 h.

### 3.2. Dynamic Creep Test

In a dynamic creep test, a repeated uniaxial stress is applied to an asphalt specimen for a number of load cycles while axial strain is measured in the same direction as the loading using linear variable differential transducers (LVDTs). The applied dynamic load used in this test was a sequence of rectangular pulses. The pulse duration was 0.5 seconds, and the rest period before the next pulse was 1.5 seconds. A static axial stress of *σ*_*s*_ = 5 kPa was applied for ten minutes to the top platen of the specimen for proper bedding, as in a static creep test. The deviator stress repeated loading was 500 kPa, and the testing temperature was set to 50°C. The failure criterion was defined as 5% of axial strain or complete failure, whichever occurred first.

### 3.3. Resonant Column Test

Characteristics of the specimens under cyclic loading were studied in two parts. The first involves the determination of the maximum shear modulus, which is evaluated on the order of 10^−4^–10^−3^% strain with a resonant column (RC) test. The RC device is the most commonly used laboratory test to measure low-strain dynamic properties of soils, concrete, and rocks. The test data analysis is described in detail by Drnevich [23] and uses the ASTM D 4015 standards.

The RC test configuration is a fixed-free system where the specimen is fixed at the bottom and free to rotate at the top at its fundamental frequency via a drive system. By measuring the motion of the free end, the velocity of the propagating wave and the degree of material damping can be derived. The shear modulus is then obtained from the derived velocity and the density of the specimen.

The test specimen is a solid cylindrical specimen with an approximate diameter-height ratio of 2. The bottom is fixed to the base of the apparatus. Sinusoidal torsional excitation is applied to the top of the specimen by an electric motor system. A torsional harmonic load of constant amplitude is applied over a range of frequencies, and the response curve (strain amplitude) is calculated. The output angular acceleration at the top of the specimen is recorded by an accelerometer. The frequency of the cyclic torque is automatically and gradually changed until the first resonance of torsional vibration is obtained. The shear wave velocity is obtained from the first-mode resonant frequency, and the shear modulus is then calculated using the shear wave velocity and the specimen density. The shear modulus and damping ratio were measured under a range of shear strains. The power is shut off at resonance (i.e., forced vibration is removed), and the material damping is determined from the free vibration decay.

The entire system is placed into a perspex chamber to apply a uniform confining pressure on the specimen using air pressure. A membrane covers the setup to prevent diffusion of air into the specimen. Identical fresh specimens were prepared using the same procedure as for the Marshall stability tests. After the 300 mm diameter cylindrical asphalt specimen had cured, it was cored into a standard size with a diameter of 70 mm for the resonant column test. The height of the specimens was approximately 140 mm.

The specimens were fixed onto the bottom pedestal using cyanoacrylate-based fast-acting adhesive. Because the strength and rigidity of the adhesive are higher than those of the asphalt, they have almost no effect on the testing data. After the adhesive was cured, the RC device was set up. Each specimen was tested in sequence with stepwise increased confining pressure. At each confining pressure, cyclic torque was applied to measure the shear modulus, *G*, and the damping ratio, *D*. The vertical pressure on the subgrade under a road is between 50 and 150 kPa when a car or loaded truck axle passes over it. Thus, the tests were conducted by employing four confining pressures of *σ*_*c*_ = 0, 50, 100, and 150 kPa. After the adjustment of each confining pressure in each test, the cell pressure was maintained for 30 minutes to allow for volume change of the specimen before the test started.

## 4. Results and Discussion

**Figure 6:**
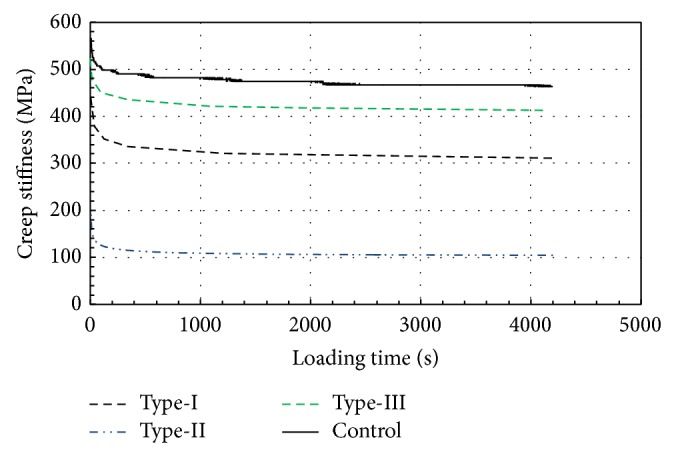
Static creep stiffness curves with respect to rubber type and time.

The corresponding static creep stiffness plotted against the loading time is shown in Figure 6. One of the outcomes seen in Figure 6 is that the static creep stiffness is not appreciably affected by the duration of loading after a certain time. However, adding rubber to the mixture gradually changes the strength properties of the specimens. This fact can be explained by considering the structure of the specimen. An asphalt specimen is composed of an assembly of aggregates and bitumen, where intergranular forces are transmitted through points of contact. When rubber is added, the resulting mixture is not always homogeneous at all contact points due to the size and shape of the rubber. Furthermore, Type-I and Type-II rubber types have relatively coarse-grained rubber crumbs, which have considerably smaller specific surface areas for a given size particle than in Type-III, and do not fully dissolve in the bitumen mixture, thereby increasing heterogeneity and void ratio. Reacted asphalt rubber materials have drastically different properties compared to unreacted asphalt rubber. The resolving of rubber increases the viscosity of bitumen and causes binding and reinforcement effects. Type-III fine powder rubber, which spread and dissolved homogeneously into the mixture, stuck to aggregate surfaces better, as seen in Figure 6.

**Figure 7:**
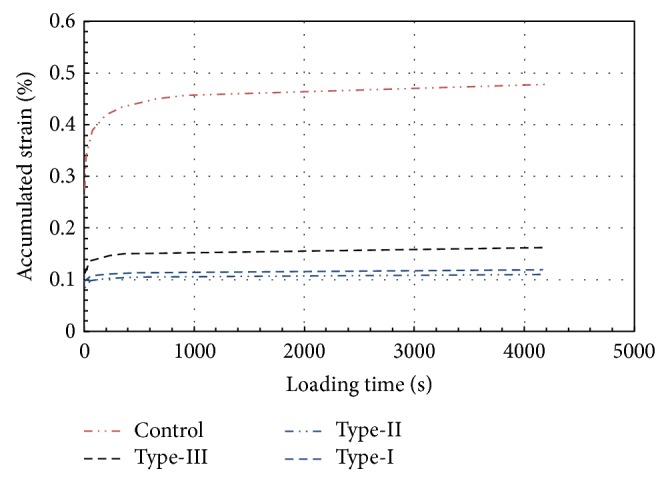
Variation of accumulated strain with respect to rubber type and time.

Static loading causes slip-down movement of aggregates, which reduces the volume of the specimen by repacking the aggregate into a denser state. When slip-down occurs, the aggregates fill the gaps in the void and do not move in the direction of loading. It is for this reason that the increase in strain is generally observed at an early stage of loading in each test, as seen in Figure 7. In Control specimen, the accumulated strain value is 0.46% at the end of the test. In rubber-modified specimens, however, the slip-down movement occurs rather easily due to the rubber between the aggregates. The accumulated strain values are approximately 0.25 of the Control specimen.

**Figure 8:**
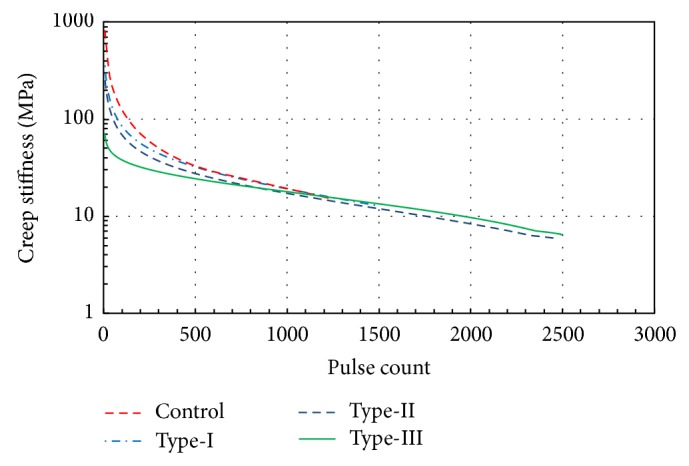
Variation in creep stiffness with respect to rubber type and pulse count.

The characteristic change in dynamic creep stiffness can be interpreted as follows: as the number of cycles increases, the dynamic creep stiffness decreases. It is interesting to note that the dynamic creep stiffness tends to decrease with an increasing number of cycles only during the first 200 cycles; thereafter, the dynamic creep stiffness reduction becomes negligibly small (Figure 8). Progressive reduction in dynamic creep stiffness is obvious for all test specimens. According to Figure 8, number of cycles for failure for unmodified and modified specimens is not same due to elastic behaviour of rubber. Failure occurred at the 2500th cycle for Type-II and Type-III whereas 1400th cycle for the Control group.

**Figure 9:**
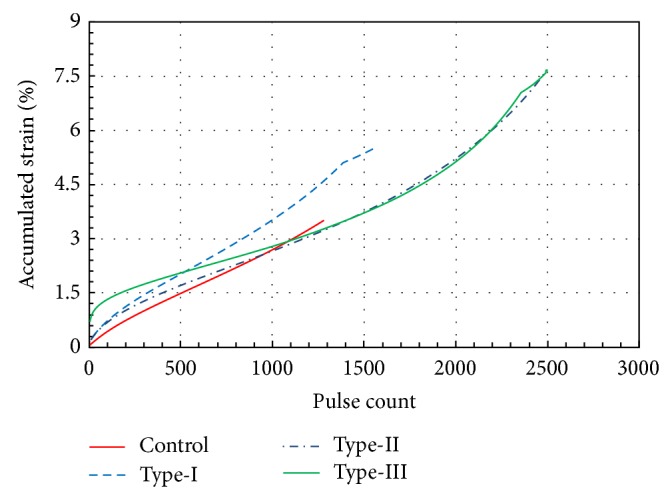
Variation in accumulated strain with respect to rubber type and pulse count.

Accordingly axial strains become considerably large in modified specimens. However, failure does not occur within the same strain. The loading continued until the magnitude of axial strain increased to a level of approximately 7.5% for Type-II and Type-III, as shown in Figure 9. The results of the tests indicate that the shear stiffness of the Control specimens gave the lowest value compared to those in the Type-I, Type-II, and Type-III, which can be attributed to the fact that when rubber and asphalt are mixed at high temperatures, such as 145–170°C, the rubber particles may swell. Swelling has been postulated to occur as a result of physical and chemical interactions between rubber particles and asphalt as well as the reaction between the asphalt and the rubber, which results in an increase in viscosity of the asphalt-rubber mixture as stated in the previous paragraphs. Furthermore, the imperfect coupling between the rubber and aggregates due to swelling causes bigger void ratios, which produce somewhat larger axial displacements than the Control specimen.

**Figure 10:**
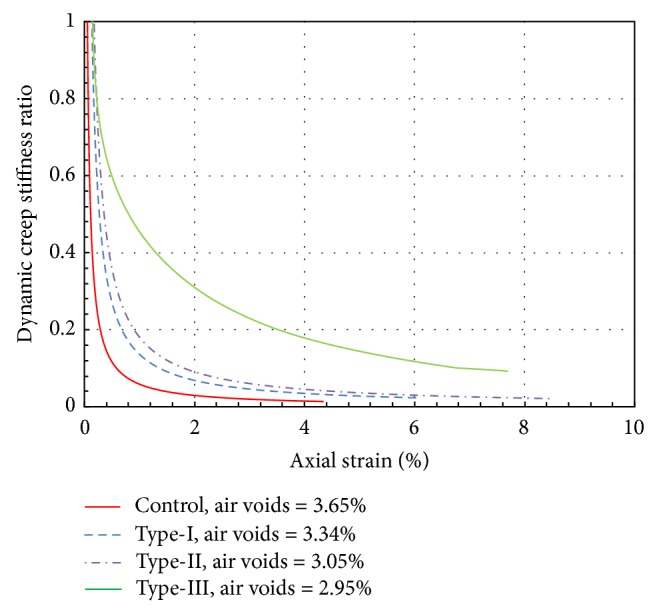
Variation in dynamic creep stiffness with respect to strain.

Figure 10 shows normalized creep stiffness plotted versus axial strain. It is interesting to note how the dynamic creep stiffness tends to change continuously with the axial strain. The normalization is made by taking the dynamic creep stiffness, CS_*d*_, at any time divided by the initial dynamic creep stiffness value, CS_*o*_. The deformation characteristic features for the Type-I, Type-II, Type-III, and Control specimens are more vividly witnessed in this plot. It is apparent from Figure 10 that the dynamic shear stiffness decreases suddenly to approximately 99% of the initial value in the Control specimen and 97% in the Type-I and Type-II specimens, for a value of 1% axial strain. The axial strain at the time of failure is 4.2% for the Control specimens and 8.5% and 6% for the Type-I and Type-II specimens, respectively. Adding rubber to the asphalt shifts the curves to the left and increases the strain value at failure. The same results are observed for Type-III, but the reduction from the original value is only 50% for 1% axial strain and does not occur as suddenly as in the other rubber-modified specimens. Moreover, the strain rate for Type-III at the time of failure is almost the same as the other specimens. The gradual shift of the curves to the left implies the increase in elastic response due to the rubber content. The graph also indicates that the rate of dynamic creep stiffness reduction with axial strain becomes greater as the void ratio decreases, implying that the aggregates are more rigidly bonded together in the Control specimen than in the modified specimens.

Due to its highly elastic nature, the responses of rubber-modified mixtures are expected to show more elastic behavior with increasing rubber content under cyclic loads. The RC test results are for shear strains less than approximately 0.0006%. It was impossible to achieve higher strains due to the torque limitation of the RC device. This limitation is satisfactory because ground vibrations produced by vehicles are assumed to induce strains in the low-amplitude range levels (i.e., less than 0.001%).

**Figure 11:**
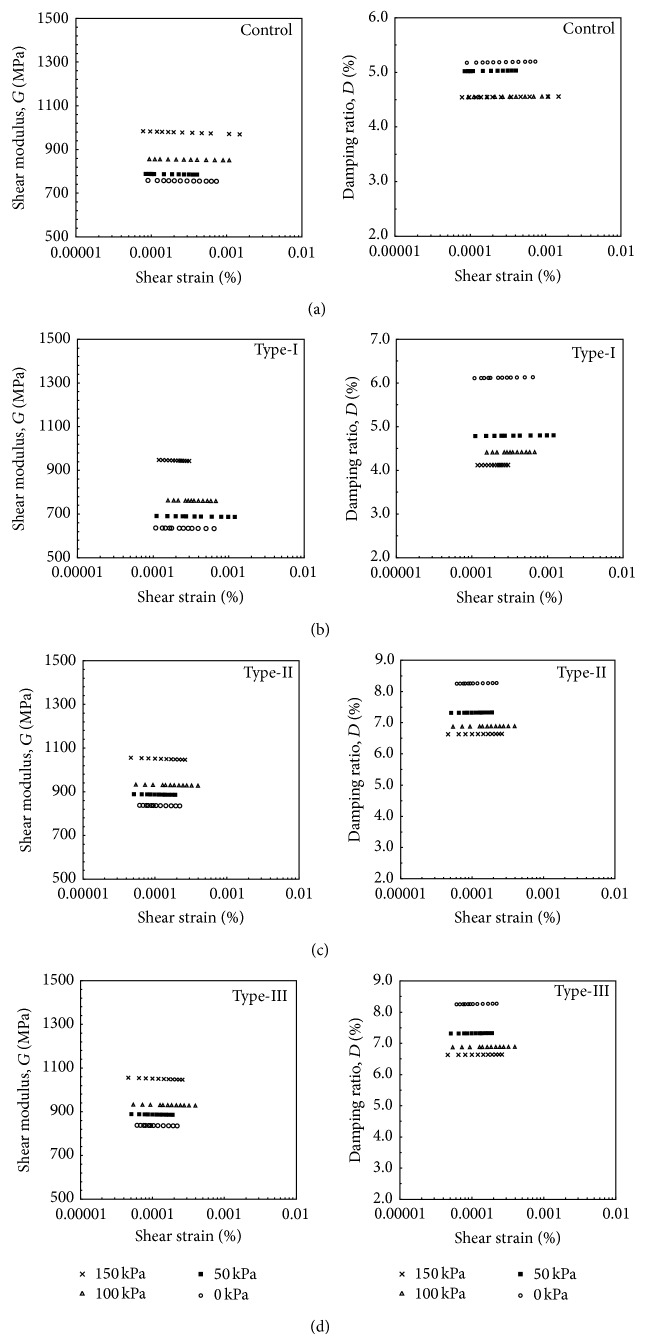
Effect of confinig stress and rubber type on shear modulus and damping ratio: (a) Control samples (b) Type-I, (c) Type-II, and (d) Type-III.

The calculated shear modulus with respect to shear strain is shown in Figure 11. As shown in Figure 11, the Type-III specimens have the largest shear modulus values compared to the Control specimens and the other rubber-modified specimens due to lower air void ratios and perfect coupling between the rubber and aggregates (Figures on the left side of Figure 11). The difference in air void ratios of rubber-modified specimens could have contributed to the increase in shear modulus; however, for the Type-I and Type-II specimens, which had the similar air void ratios, the stiffness did increase.

The shear modulus of rubber-modified specimens was somewhat lower and the damping ratio was considerably higher than those of the Control specimens at corresponding confining pressures. Thus, it can be concluded that adding a certain amount of rubber to an asphalt mix can slightly decrease the shear stiffness, whereas significantly increases damping. Increasing the confining pressure from 0 to 150 kPa increased the shear modulus by approximately 20%. The initial shear modulus increases noticeably in all cases with an increase in confining pressure; however, the rate of increase becomes small after the first incremental stage (from 0 to 50 kPa) and diminishes after 100 kPa. The results agree with the characteristic properties of asphalt obtained from other tests such as the Marshall stability test.

The damping ratio is an important characteristic of a material because it indicates how much vibration energy is absorbed during a vibration cycle. If a material has a high damping ratio, attenuation of vibration will also be high. On the other hand, it is not easy to define true material damping, but it is common to express the damping of real materials in terms of their equivalent viscous damping ratios. The viscous damping ratio, *D*, is measured in the resonant column test from the shape of a free vibration decay curve. This curve is measured using the accelerometer mounted on the resonant column drive plate. A sinusoidal wave is applied to the soil, after which the excitation is shut off so the resulting free vibrations can be measured.

The value of the damping ratio obtained in the same test series is shown on the right side of Figure 11 plotted against the shear strain levels. In all of the figures, the damping ratio increases slightly with increasing shear strain, irrespective of whether the specimen has been modified and also independently of rubber size. It is also obvious from the figures that the damping ratio increases due to the confining stress becoming more pronounced with increasing rubber content and decreasing rubber particle size.

The shear modulus decreased by 20% in the Type-I and Type-II specimens compared to the Control specimen. However, the greatest increase in shear stiffness, 10% compared to the Control specimen, occurred in the Type-III specimens. On the other hand, the damping increased by 30% in the Type-I and Type-II specimens and 40% in the Type-III specimens. The shear modulus of the specimens decreased slightly with shear strain in all tests, as expected. The shear modulus of the Control specimens was somewhat higher than that of specimens Type-I, Type-II, and Type-III at the corresponding confining pressures. The weak interaction between the rubber particle surface and the asphalt changes under static or dynamic loads. Increased specific surface increases the reaction rate with hot asphalt, as in the case of the Type-III specimens.

The aggregates in the asphalt concrete are extremely stiff and therefore dissipate very little energy in particle deformation. In contrast, the rubber consumes energy through deformation of the rubber particles themselves. It is seen that, no matter the size of the crumb rubber, the static and dynamic stiffness decrease with any proportion of rubber in the asphalt. However, modified asphalt improves longevity compared to the Control specimens.

## 5. Conclusions 

Creep stiffness is dependent on the type of loading, regardless if it is static or dynamic. It is highly dependent on the strain level at which the creep value is determined and also strongly influenced by the rubber particle size and texture. The particle size affects the shear modulus and damping ratio.

It can be concluded that the rubber-modified asphalt concrete mitigates vibrations generated by traffic loading and results in reduced damage from cyclic straining. The use of polymer-modified bitumen provides improved longevity and marked whole-life cost benefits, increasing the sustainability of pavements.

## Figures and Tables

**Table 1:** Physical characteristics of the aggregates used in the tests.

Properties	Test values	Standards
Specific gravity of coarse aggregate, 25°C, g/cm^3^	2.62	ASTM C127-07
Water absorption of coarse aggregate, %	0.23	ASTM C127-07
Specific gravity of fine aggregate, 25°C, g/cm^3^	2.622	ASTM C128-07a
Water absorption of fine aggregate, %	1.04	ASTM C128-07a
Specific gravity of filler, 25°C, g/cm^3^	2.708	ASTM C128-07a
Los Angeles wearing test, %	28.91	ASTM C535-09
Freezing and thawing test, %	5.467	ASTM C1646-08a
Bitumen absorption, %	0.14	ASTM D4469-01

**Table 2:** Test results of the mix design.

Properties of the mix design	
Unit weight, kg/cm^3^	2413.36
Marshall stability, N	16500
Void, %	3.2
Voids filled with bitumen, %	76.4
Flow, mm	1.82
Coarse aggregate (%)	36.5
Fine aggregate (%)	56.5
Filler (%)	7

**Table 3:** Properties of bitumen.

Properties	Values	Related standards
Penetration at 25°C, 1/10 mm	57.3	ASTM D 5-06e1
Ductility at 25°C, cm	>100	ASTM D 113-99
Loss on heating, %	0.17	ASTM D 6-95
Specific gravity at 25°C, gr/cm^3^	1.035	ASTM D 70-03
Softening point, °C	48.0	ASTM D 36-09
Flash point, °C	308	ASTM D 92-02b
Elastic recovery, % (25°C)	2.95	ASTM D 6084-06
